# Comparative transcriptomic and metabolomic analyses of carotenoid biosynthesis reveal the basis of white petal color in *Brassica napus*

**DOI:** 10.1007/s00425-020-03536-6

**Published:** 2021-01-02

**Authors:** Ledong Jia, Junsheng Wang, Rui Wang, Mouzheng Duan, Cailin Qiao, Xue Chen, Guoqiang Ma, Xintong Zhou, Meichen Zhu, Fuyu Jing, Shengsen Zhang, Cunmin Qu, Jiana Li

**Affiliations:** 1 Chongqing Rapeseed Engineering Research Center, College of Agronomy and Biotechnology, Southwest University, Chongqing, 400715 China; 2 Academy of Agricultural Sciences, Southwest University, Chongqing, 400715 China; 3 College of Life Science and Agronomy, Zhoukou Normal University, Zhoukou, 466001 China

**Keywords:** *BnNCED4*, Carotenoid biosynthetic pathway, Carotenoid cleavage dioxygenases, Lutein, RNA-seq, Zeaxanthin

## Abstract

**Main conclusion:**

The molecular mechanism underlying white petal color in *Brassica napus* was revealed by transcriptomic and metabolomic analyses.

**Abstract:**

Rapeseed (*Brassica napus* L.) is one of the most important oilseed crops worldwide, but the mechanisms underlying flower color in this crop are known less. Here, we performed metabolomic and transcriptomic analyses of the yellow-flowered rapeseed cultivar ‘Zhongshuang 11’ (ZS11) and the white-flowered inbred line ‘White Petal’ (WP). The total carotenoid contents were 1.778-fold and 1.969-fold higher in ZS11 vs. WP petals at stages S2 and S4, respectively. Our findings suggest that white petal color in WP flowers is primarily due to decreased lutein and zeaxanthin contents. Transcriptome analysis revealed 10,116 differentially expressed genes with a fourfold or greater change in expression (*P*-value less than 0.001) in WP vs. ZS11 petals, including 1,209 genes that were differentially expressed at four different stages and 20 genes in the carotenoid metabolism pathway. *BnNCED4b*, encoding a protein involved in carotenoid degradation, was expressed at abnormally high levels in WP petals, suggesting it might play a key role in white petal formation. The results of qRT-PCR were consistent with the transcriptome data. The results of this study provide important insights into the molecular mechanisms of the carotenoid metabolic pathway in rapeseed petals, and the candidate genes identified in this study provide a resource for the creation of new *B. napus* germplasms with different petal colors.

**Supplementary Information:**

The online version contains supplementary material available at 10.1007/s00425-020-03536-6.

## Introduction

Carotenoids, among the most important pigments in plants, are composed of various combinations of isoprene units, including several oxygen-free carotenes and oxygen-containing xanthophylls (Alkema and Seager 1982). Carotenoids are found ubiquitously in plants, as they are essential components of photosystems, and they confer yellow-to-red coloration to flowers and fruits (Tanaka et al. 2008). In addition to their essential roles in photosynthesis (Young and Britton 2012), carotenoids provide the substrates needed for the biosynthesis of the plant growth regulator abscisic acid (ABA) (Nambara and Marion-Poll 2005) as well as other hormones (Auldridge et al. 2006). More than 700 naturally occurring carotenoids have been identified, eight of which are currently produced synthetically on an industrial scale (Ernst 2002). Carotenoids also play important roles in human nutrition and health, providing a source of provitamin A and showing antioxidant and anti-cancer activities (Rao and Rao 2007). Some carotenoids are used as food colorants and as additives in the cosmetics and pharmaceutical industries (Tanaka et al. 2008).

The carotenoid biosynthetic pathway has been well characterized in many plant species (Cunningham and Gantt 1998), including Arabidopsis (*Arabidopsis thaliana*) (Von Lintig et al. 1997), tomato (*Solanum lycopersicum*) (Giuliano et al. 1993), maize (*Zea mays*) (Vallabhaneni and Wurtzel 2009), and rice (*Oryza sativa*) (Beyer et al. 2002). Carotenoids are synthesized from geranylgeranyl diphosphate (GGPP) by the sequential addition of three isopentenyl pyrophosphate (IPP) molecules to dimethylallyl diphosphate (DMAPP), of which IPP and DMAPP are both derived from the methylerythritol 4-phosphate (MEP) pathway. IPP is isomerized to DMAPP by the enzyme IPP isomerase (encoded by *IPI* genes) (Cunningham and Gantt 1998; Hirschberg 2001; Tanaka et al. 2008). GGPS catalyzes the conversion of IPP and DMAPP to GGPP. The first C40 carotenoid, phytoene, is produced by the head-to-head coupling of two GGPP molecules in a reaction catalyzed by phytoene synthase (PSY). Phytofluene is then synthesized from phytoene by two desaturases and two isomerases, including phytoene desaturase (PDS), 15-cis-zeta-carotene isomerase (Z-ISO), zeta-carotene desaturase (ZDS), and carotenoid isomerase (CRTISO). Subsequently, lycopene ε-cyclase (LCYE) and lycopene β-cyclase (LCYB) catalyze the conversion of phytofluene to δ-carotene and α-carotene (α-branch), γ-carotene, and β-carotene (β-branch). α-carotene is then converted to lutein via a process catalyzed by ε-ring carotene hydroxylase (CHYE) and β-ring carotene hydroxylase (CHYB), and β-carotene is converted to β-cryptoxanthin and zeaxanthin via a process catalyzed by β-carotene hydroxylase (BCH). Subsequently, zeaxanthin epoxidase (ZEP) catalyzes the synthesis of antheraxanthin and violaxanthin from zeaxanthin, and neoxanthin synthetase (NXS) catalyzes the conversion of violaxanthin to neoxanthin. Finally, neoxanthin is degraded into the precursors of ABA via a process catalyzed by carotenoid cleavage dioxygenases (CCD) and nine-cis-epoxycarotenoid dioxygenases (NCED).

The carotenoid biosynthetic pathway is regulated by transcription factors from several different families (Stanley and Yuan 2019), including the R2R3-MYB, MADS-box, NAC, bHLH, SBP-box, AP2/ERF, HD-ZIP, NF-Y, and WRKY families, among others. In tobacco, knockdown of *MYB305* by RNAi resulted in reduced β-carotene levels (Liu and Thornburg 2012). Loss-of-function mutations in *RCP1* (R2R3-MYB) led to the downregulation of all carotenoid biosynthetic genes and reduced carotenoid contents in RNAi *Mimulus lewisii* flowers (Sagawa et al. 2016). The R2R3-MYB transcription factor CrMYB68 negatively regulates *CrBCH2* and *CrNCED5* expression and directly regulates the transformation of α-branch and β-branch carotenoids (Zhu et al. 2017a, b). The accumulation of carotenoids and the expression of *PHYTOENE SYNTHETASE1* were enhanced in *SlMADS1* RNAi tomato fruits (Dong et al. 2013) but reduced in *SlCMB1* (encoding a MADS-box transcription factor) RNAi tomato fruits (Zhang et al. 2018). Fu et al. (2016) suggested that CpNAC1 positively regulates carotenoid biosynthesis during papaya fruit ripening. Finally, tomato plants overexpressing *SlPRE2* (encoding a bHLH transcription factor) produced light-green unripened fruits and yellowing ripened fruits with reduced levels of chlorophyll and carotenoids, respectively, in their pericarps (Zhu et al. 2017a, b).

Rapeseed flowers typically have yellow petals. To date, as the source of a popular ornamental flower, only a few mutations have been shown to affect petal color in rapeseed, leading to petal colors such as golden yellow, orange yellow, creamy white, white, and so on. The carotenoid cleavage dioxygenase 4 gene *BnaC3.CCD4* contributes to the loss of flower color in white-flowered *B*. *napus* lines (Zhang et al. 2015). Yao et al. (2017) identified two major candidate intervals of the orange petal color gene *Bnpc1* on chromosome C09 of *B. napus* by whole-genome re-sequencing and bulked segregation analysis of backcrossing (BC4) individuals. Furthermore, total carotenoid contents in rapeseed sprouts are affected by light treatment, and differences in flower color were ascribed to higher carotenoid contents in yellow-flowered cultivars compared to white-flowered cultivars (Groenbaek et al. 2018). However, how carotenoid accumulation in rapeseed is regulated is unclear, and the detailed molecular mechanism underlying white flower formation in rapeseed remains to be uncovered.

Here, we compared the yellow-flowered rapeseed cultivar ‘ZS11’, which is widely grown in China, with the petal color mutant line ‘WP’. We analyzed the carotenoid biosynthetic pathway in petals at stage 2 (S2; with pale yellow petals) and S4 (with white petals) in WP and in petals at the corresponding stages in ZS11. We identified the components of the carotenoid biosynthetic pathway in the four pools of petals via multiple reaction monitoring (MRM) using the absolute quantification method. We also analyzed the transcriptomes of petals at the B5 (5 mm long buds), B7 (7 mm long buds), S1 (open buds before the petals spread), and S3 (the petals had just fully spread) stages in WP and the B5, B8 (8 mm long buds), S1, and S3 stages in ZS11 and measured the expression of the carotenoid biosynthetic genes by transcriptomic analysis and quantitative reverse-transcription PCR (qRT-PCR). Finally, we systematically analyzed the regulatory genes of the carotenoid biosynthetic pathway. The results of this study reveal comprehensive changes in the metabolomic and transcriptomic networks underlying the formation of white and yellow petals in rapeseed at different developmental stages, providing important insights into the molecular mechanisms of the carotenoid biosynthetic pathway. Moreover, the candidate genes identified in this study will provide a resource for the creation of new flower colors germplasms in *B. napus*.

## Materials and methods

### Plant materials

**Fig. 1:**
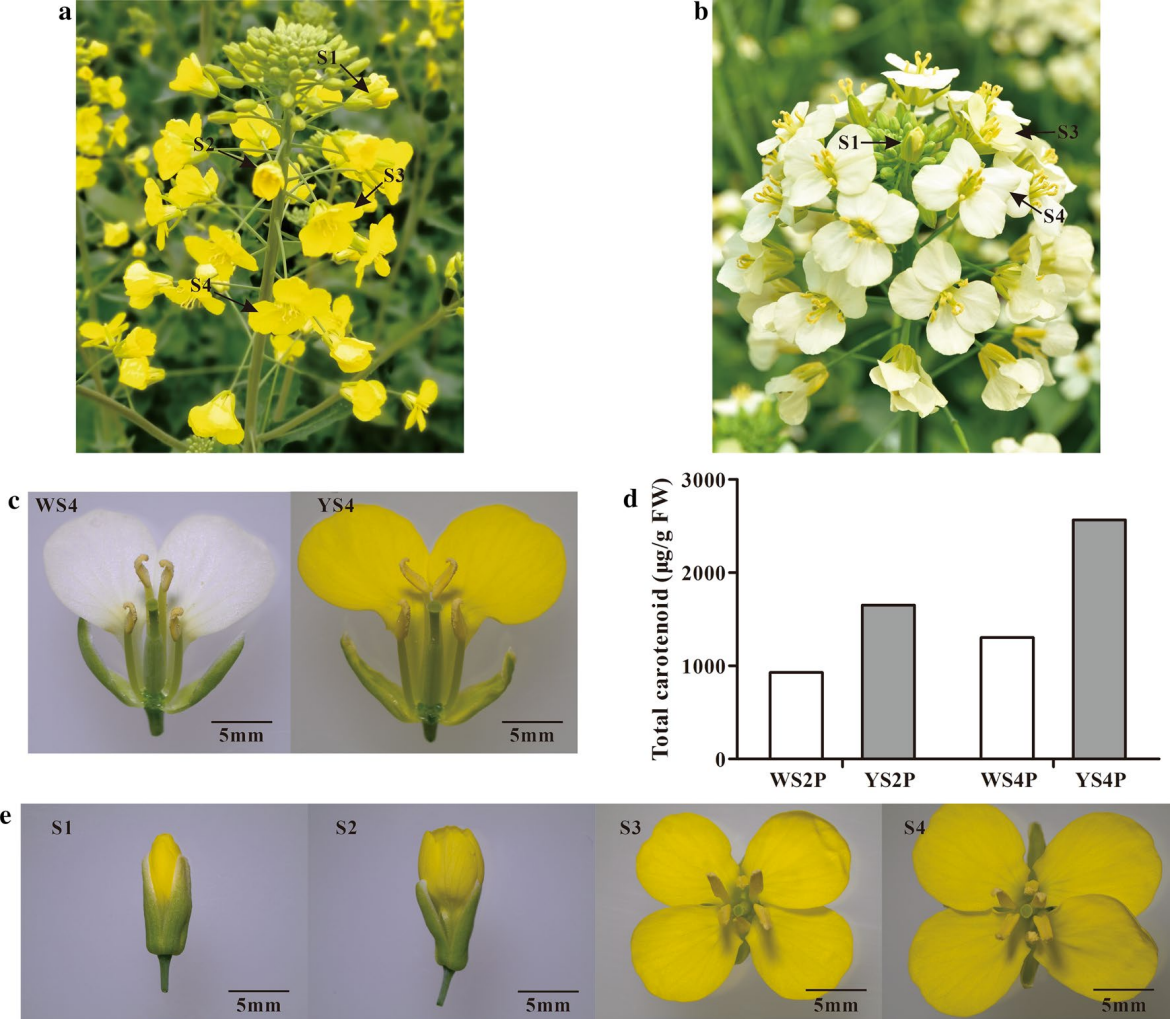
Phenotype of WP and ZS11 and the total carotenoid content of petals at S2 and S4 stages. **a** Inflorescence of ZS11 cultivar in flourishing florescence. **b** Inflorescence of WP cultivar in flourishing florescence. **c** Flowers of WP and ZS11 at S4 stage. **d** Total carotenoid content of WP and ZS11 petals at S2 and S4 stages. **e** Flowers of ZS11 at S1–S4 stages

‘WP’ is a stable white-flowered inbred line derived from a distant hybridization between the normal yellow-flowered rapeseed and the white-flowered *B*. *alboglabra*, and was selected from progenies of the advanced backcross population with the white-flowered individual as the donor parent and the normal yellow-flowered rapeseed as the recurrent parent. The common yellow-flowered rapeseed cultivar ‘ZS11’ and the white-flowered inbred line ‘WP’ were cultivated in the experimental station at the Rapeseed Engineering Research Center of Southwest University in Beibei (29º21ʹ N, 106º21′ S), Chongqing city, China. White and yellow petals at different stages were collected for metabolomics analysis in March 2018, and samples for RNA-sequencing (RNA-Seq) and qRT-PCR validation were collected in March 2019. Petals from flowers at the bud phase and the flowering phase were sampled from both lines, including the following: petals from 5 mm long WP buds (WB5P); petals from 5 mm long ZS11 buds (YB5P); petals from 7 mm long (the maximum length before flowering) WP buds (WB7P); petals from 8 mm long (the maximum length before flowering) ZS11 buds (YB8P); petals from open WP buds before the petals spread (WS1P); petals from ZS11 open buds before the petals spread (YS1P); petals from WP flowers when the petals were half spread (WS2P); petals from ZS11 flowers when the petals were half spread (YS2P); petals from WP flowers when the petals had just fully spread (WS3P); petals from ZS11 flowers when the petals had just fully spread (YS3P); petals from WP flowers two days after the petals had fully spread (WS4P); and petals from ZS11 flowers two days after the petals had fully spread (YS4P). The ZS11 petals at stages S1-S4 are shown in Fig. 1e. Each petal sample was collected from 30 randomly selected plants in the same block, with ten rows and eight plants per row. The petals were separated, collected, immediately frozen in liquid nitrogen, and stored at -80℃ until use. The chromaticity values of S4 stage petals from ZS11 and WP (Fig. 1c) were determined using a YS3060 Grating Spectrophotometer (Shenzhen Threenh Technology Co., Ltd.) with a measuring aperture of 8 mm; the assays were repeated three times.

### Extraction and quantification of carotenoids

WS2P, WS4P, YS2P, and YS4P petal samples were freeze-dried and ground into a fine powder using a mixer mill (MM 400, Retsch) with zirconia beads for 1 min at 30 Hz. Each sample (100 mg) was weighed and extracted with n-hexane:acetone:ethanol (2:1:1, by vol.) with 0.01% butylated hydroxytoluene (BHT) (g/mL) at room temperature. The sample was vortexed for 30 s, subjected to ultrasound-assisted extraction for 20 min at room temperature, and centrifuged for 5 min at 10,000 g at room temperature. After repeating these steps, the two supernatants were collected and evaporated to dryness under a nitrogen gas stream. The residue was dissolved in 200 μL mixing solution (acetonitrile: methanol [3:1, v/v]: methyl tert-butyl ether [85:15, v/v]), vortexed for 30 s, subjected to ultrasound-assisted extraction for 2 min, and centrifuged for 5 min at 10,000 g. The supernatants were absorbed and filtered (0.22 µm pore size) prior to LC–MS analysis.

Each 5 μL supernatant sample was injected into a HPLC system (Shim-pack UFLC Shimadzu CBM30A system, www.shimadzu.com.cn/) equipped with a C30 column (3 µm, 2 mm × 100 mm). HPLC analysis was performed using a binary solvent system comprising acetonitrile:methanol (3:1, v/v) (0.01% BHT) as mobile phase A and methyl tert-butyl ether (0.01% BHT) as mobile phase B. The A:B (v/v) gradient program was 85:15 at 0 min, 75:25 at 2.0 min, 40:60 at 2.5 min, 5:95 at 3.0 min, 5:95 at 4.0 min, 85:15 at 4.1 min, 85:15 at 6.0 min. The flow rate was kept at 0.8 mL/min, and the column temperature was maintained at 28 °C. The effluent was alternatively connected to an ESI-triple quadrupole-linear ion trap (Q TRAP)-MS system. Linear ion trap (LIT) and triple quadrupole (QQQ) scans were acquired on an API 6500 Q TRAP LC/MS/MS system equipped with an APCI Turbo Ion-Spray interface operating in negative ion mode and controlled by Analyst 1.6.3 software (AB Sciex). The APCI source operation parameters were as follows: ion source, turbo spray; source temperature, 350 °C; ion spray voltage (IS) voltage, 3.5 kV; the ion source gas I (GSI), gas II (GSII), and curtain gas (CUR) were set at 55, 60, and 25.0 psi, respectively; and the collision gas (CAD) was set at medium. DP and CE for individual MRM transitions were performed with further DP and CE optimization. A specific set of MRM transitions was monitored for each period according to the plant hormones eluted within this period. The MRM for each line was performed in triplicate. Three spears were used for each repeat.

Standard solutions of different concentrations of plant carotenoids were prepared, and the peak intensity data for the corresponding quantitative signals for each concentration standard were obtained to construct standard curves for different carotenoids, with the concentrations of carotenoid standards (ng/mL) listed on the abscissa and peak areas listed on the ordinate. The peak areas of absorbance (carotenoids) in all samples were calculated based on the linear equation of the standard curve, and the absolute carotenoid contents in test samples were calculated using the formula: Carotenoid content in samples (μg/g) = B*C/1000/D, where B is the area of the integrated value of the absorbance peak in the sample substituted for the concentration from the standard curve (μg/mL); C is the volume of solution (μL); and D is the sample quantity (g). The carotenoid metabolome was analyzed by Metware Biotechnology Co., Ltd. (Wuhan, China).

### RNA-seq and quality control

Total RNA was extracted from WB5P, YB5P, WB7P, YB8P, WS1P, YS1P, WS3P, and YS3P petal samples using an EZ-10 Total RNA Mini-Preps Kit (Sango Biotech, Shanghai, China); three biological repeats were prepared for each sample. Sequencing library construction and transcriptome sequencing were performed by Novogene Bioinformatics Technology Co., Ltd. (Beijing, China). The mRNA was purified from total RNA using poly-T oligo-attached magnetic beads, and the library quality was assessed using the Agilent Bioanalyzer 2100 system. After cluster generation, the library preparations were sequenced on an Illumina HiSeq™ 4000 platform, and 150 bp paired-end reads were generated. Raw data (raw reads) in Fastq format were processed by removing reads containing adapters, reads containing poly-N, and low-quality reads, and the downstream analyses were performed on the remaining high-quality data. The raw sequencing data reported in this study have been deposited in the Genome Sequence Archive (Wang et al. 2017) in the BIG Data Center (Zhang et al. 2019), Beijing Institute of Genomics (BIG), Chinese Academy of Sciences under accession number CRA002052 and are publicly accessible at https://bigd.big.ac.cn/gsa.

## RNA-seq analysis

### Raw data filtration and mapping

The raw data were checked using FastQC software, and the base quality values of the sequencing data were obtained, including sequence length, sequence number, GC content, base quality, and so on. Quality filtering was carried out using Trimmomatic 0.33 software. The reference genome of the *Brassica napus* cultivar ‘Darmor-*bzh*’ was downloaded from the French *B. napus* genome database (http://www.genoscope.cns.fr/brassicanapus/) (Chalhoub et al. 2014), and the clean data were compared with the reference genome using HiSat 2.0.4 software. Cufflinks 2.2.1 was used to calculate gene expression levels, and Samtools 1.2 was used to sort and index the final BAM files. The cuffquant and cuffnorm programs in the Cufflinks software package were used to count the reads mapped to each gene. The FPKM value of each gene was calculated based on the length of the gene and the reads count mapped to the gene. FPKM, the expected number of Fragments Per Kilobase of transcript sequence per Million base pairs sequenced, simultaneously considers the effects of sequencing depth and gene length for each read count and is currently the most commonly used method for estimating gene expression levels.

### Identification of differentially expressed genes

Differential gene expression analysis of two groups (three biological replicates per group) was performed using the DESeq2 R package (1.16.1). DESeq2 provides statistical routines for determining differential expression in digital gene expression data using a model based on the negative binomial distribution. The resulting *P*-values were adjusted using the Benjamini and Hochberg’s approach for controlling the false discovery rate. Genes with an adjusted *P*-value < 0.001 detected by DESeq2 and absolute fold-change of 4 were considered to be significant differentially expressed genes (DEGs). Gene Ontology (GO) and Kyoto Encyclopedia of Genes and Genomes (KEGG) enrichment analyses of the DEGs were performed using the OmicShare tools, a free online platform for data analysis (https://www.omicshare.com/tools). GO terms and KEGG pathways with *P*-value < 0.05 were considered to be significantly enriched by the DEGs, and the enrichment results were plotted using online software available on OmicShare.

### qRT-PCR validation

To validate the transcriptome data and characterize the DEGs between WP and ZS11 petals, the expression levels of differentially expressed carotenoid biosynthetic genes were determined simultaneously, including *BnPSYc*, *BnPSYg*, *BnPDS1a*, *BnPDS1b*, *BnPDS1c*, *BnPDS1d*, *BnZ-ISOa*, *BnZ-ISOb*, *BnCrtISOa*, *BnCrtISOb*, *BnLYCEd*, *BnLYCBb*, *BnBCH1b*, *BnBCH1d*, *BnBCH2b*, *BnZEPa*, *BnVDEb*, *BnNCED3c*, *BnNCED4b*, and *BnNCED4c*. The cDNAs were synthesized according to the manufacturer’s protocol of PrimeScript™ RT reagent Kit with gDNA Eraser (Perfect Real Time) (Takara, Dalian, China) and used as templates for qRT-PCR analysis with primers designed based on the reference *B. napus* gene sequences using Primer Premier 5.0. PCR was conducted using SYBR Premix Ex Taq II (Perfect Real Time) (TaKaRa) in a typical 10 μL PCR mixture that included 5 μL of SYBR Premix Ex Taq II, 1 μL (100 ng) of template cDNA, 3 μL ddH_2_O, and 0.5 μL of each PCR primer (10 μM). Cycling conditions were 95 °C for 2 min, followed by 40 cycles of 95 °C for 3 s (denaturation), and 60 °C for 30 s (annealing and extension). The melting curve for each PCR amplicon was obtained under the following conditions: 95 °C for 5 s and 65 °C for 5 s, followed by a constant increase in temperature from 65 °C to 95 °C at an increment of 0.5 °C/cycle. Samples were run on the Bio-Rad CFX96 Real-Time System (Bio-Rad, Hercules, CA, USA). Relative expression of the target genes was analyzed by the 2^−ΔΔCt^ method using *BnACTIN7* (AF111812; forward primer: 5′-TGGGTTTGCTGGTGACGAT-3′, reverse primer: 5′-TGCCTAGGACGACCAACAATACT-3′) as an internal control (Sharma et al. 2007). All samples were amplified in triplicate from the same total RNA preparation, and the mean value was used for further analysis.

## Results

### Carotenoid contents in ZS11 and WP petals

**Fig. 2:**
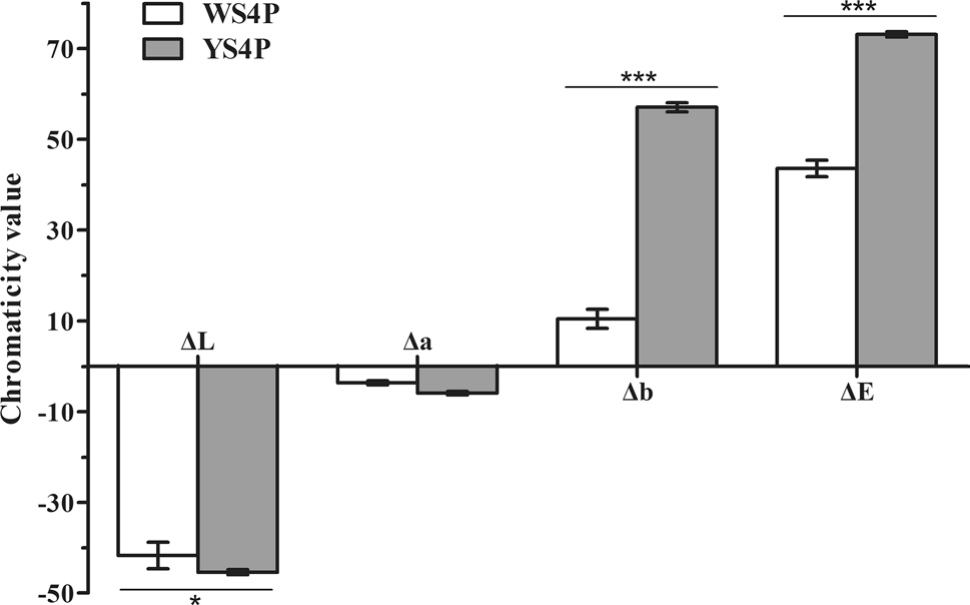
Chromaticity value of the WP and ZS11 petals at S4 stage. ΔL, the color brightness values; Δ*a*, the red colorimetric values; Δ*b*, the yellow colorimetric values; ΔE, total chromaticity values. Values are means ± SD of three experiments. Error bars indicate SEs. The asterisks above or under the columns indicate significant differences between WS4P and YS4P for different colorimetric values (t test, *P < 0.05, ***P < 0.001)

There was no significant difference in flower morphology between ZS11 and WP, except for changes in petal color during development (Fig. 1a, b). ZS11 petals retained their yellow color throughout petal development (Fig. 1a). By contrast, WP petals were pale yellow at S1, began to fade, and had turned white by S4 (Fig. 1b). We measured the chromaticity values of WP and ZS11 petals at S4 using the YS3060 Grating Spectrophotometer (Fig. 1c). As shown in Fig. 2, the color brightness (ΔL) values of WP petals were significantly greater than those of ZS11 petals (*P*-value < 0.05); in addition, the yellow colorimetric values (Δb) and total chromaticity values (ΔE) of WP and ZS11 petals were significantly different (*P*-value < 0.001). These results indicate that the major difference between WP and ZS11 petals is the variation in yellow colorimetric values during petal development.

We collected WP and ZS11 petals at S2 and S4 and subjected them to metabolomic analysis of carotenoids. Among the 13 carotenoids examined, ten were identified in the samples (Table 1), and no other products of intermediate metabolism were detected. At S2, the total carotenoid contents of WP and ZS11 petals were 928.450 and 1650.216 μg/g, respectively, i.e., 1.777-fold higher in ZS11 vs. WP. At S4, the total carotenoid contents of WP and ZS11 petals were 1303.047 and 2565.469 μg/g, respectively, i.e., 1.969-fold higher in ZS11 vs. WP (Fig. 1d; Table 1). These results indicate that the difference in petal color between WP and ZS11 is mainly due to differences in total carotenoid contents. Phytoene, lutein, and zeaxanthin were the major carotenoids at S2 and S4 in both WP and ZS11 petals, and phytofluene was also a major constituent of WP petals at S4 (Supplementary Fig. S1); the levels of all these compounds were > 60 μg/g (Table 1). From S2 to S4, the total carotenoid contents in WP and ZS11 petals increased 40.346% and 55.463%, respectively. However, the lutein and zeaxanthin (xanthophylls, which appear yellow) contents in WP petals significantly decreased (by 70.300 and 11.156 μg/g, respectively) during this period, whereas the lutein content in ZS11 petals significantly decreased (by 132.225 μg/g) during this time.

**Table 1:** Major carotenoid constituents in the petals of WP and ZS11 at S2 and S4 stages

Comparison group	Metabolite	Content (μg/g)				Fold change
		WP	Percent (%)	YP	Percent (%)	YP/WP
WS2P vs YS2P	Phytoene	630.130	67.869%	1209.791	73.311%	1.920
	Lycopene	3.262	0.351%	3.255	0.197%	0.998
	Phytofluene	28.375	3.056%	9.162	0.555%	0.323
	δ-Carotene	0.704	0.076%	0.584	0.035%	0.830
	Lutein	162.745	17.529%	284.577	17.245%	1.749
	γ-Carotene	3.090	0.333%	1.276	0.077%	0.413
	β-Carotene	2.142	0.231%	2.019	0.122%	0.943
	Zeaxanthin	77.725	8.372%	118.555	7.184%	1.525
	Violaxanthin	–	–	11.101	0.673%	/
	Neoxanthin	20.278	2.184%	13.150	0.797%	0.649
	Total	928.450	100%	1650.216	100%	1.777
WS4P vs YS4P	Phytoene	904.837	69.440%	2138.931	83.374%	2.364
	Lycopene	3.176	0.244%	3.010	0.117%	0.948
	Phytofluene	207.325	15.911%	13.496	0.526%	0.065
	δ-Carotene	0.400	0.031%	0.401	0.016%	1.003
	Lutein	90.446	6.941%	152.352	5.939%	1.684
	γ-Carotene	1.023	0.078%	1.299	0.051%	1.271
	β-Carotene	2.116	0.162%	2.044	0.080%	0.966
	Zeaxanthin	66.569	5.109%	228.348	8.901%	3.430
	Violaxanthin	–	–	13.891	0.541%	/
	Neoxanthin	27.157	2.084%	11.696	0.456%	0.431
	Total	1303.047	100%	2565.469	100%	1.969

(–) means the carotenoid was not detected in the petals

At S2, the total lutein plus zeaxanthin contents in WP and ZS11 petals were 240.470 and 403.132 μg/g, respectively, i.e., 1.676-fold higher in ZS11 than in WP. At S4, the total lutein plus zeaxanthin contents in WP and ZS11 petals were 157.015 and 380.700 μg/g, respectively, i.e., 2.425-fold higher in ZS11 than in WP. From S2 to S4, the total lutein plus zeaxanthin content in WP petals decreased by 34.705% (83.455 μg/g), whereas that in ZS11 petals decreased by only 5.564% (22.432 μg/g). These results indicate that the lighter color of WP petals is mainly due to lower carotenoid contents compared to ZS11 petals at the same stage. In addition, WP petals faded from pale yellow to white from S2 to S4 primarily due to a decrease in total lutein plus zeaxanthin contents.

### RNA-seq analysis of WP and ZS11 petals

The major characteristics of the 24 cDNA libraries created from three independent biological replicates of WP and ZS11 petals at four stages (B5, B7 or B8, S1, and S3) are summarized in Supplementary Table S1. After removing adaptor sequences, ambiguous nucleotides, and low-quality sequences, 47.1 Gb and 44.3 Gb of original data were obtained in the WP and ZS11 libraries, and the Q20 (sequencing error rate < 1%) and Q30 (sequencing error rate < 0.1%) values were ≥ 99.87% and 93.05%, respectively. The G + C percentage was 46%. After filtering by Trimmomatic, 677,351,694 and 639,227,560 reads were obtained in the WP and ZS11 libraries, respectively, with Q30 values ≥ 97.51%. Using Hisat2 to compare these reads with the reference genome of *B. napus* (Chalhoub et al. 2014), the comparison rate of all reads was ≥ 91.65%, and the perfect matching rate was ≥ 87.57% (Supplementary Table S2). The raw sequencing data reported in this study have been deposited in the Genome Sequence Archive in the BIG Data Center.

### Differential expression analysis of petals at four stages of development

**Fig. 3:**
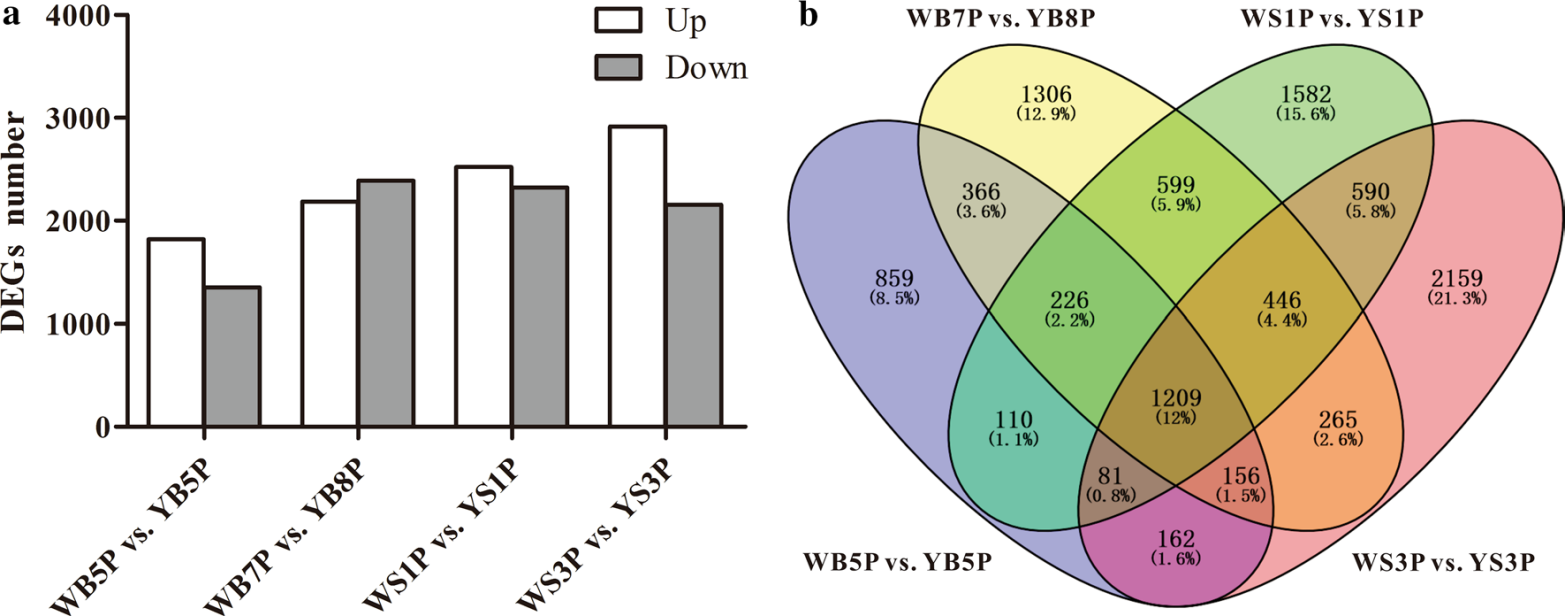
DEGs in the four comparison groups: WB5P vs. YB5P, WB7P vs. YB8P, WS1P vs. YS1P and WS3P vs. YS3P. **a** The numbers of up- and down-regulated genes in WP petals compared with ZS11 petals in the four comparison groups. **b** Venn diagram of the DEGs in the four comparison groups

We identified 3,169, 4,573, 4,843, and 5,068 DEGs in WB5P vs. YB5P, WB7P vs. YB8P, WS1P vs. YS1P, and WS3P vs. YS3P, respectively. Compared to ZS11 petals, 1,817, 2,184, 2,521, and 2,914 genes were upregulated in WP and 1,352, 2,389, 2,322, and 2,154 genes were downregulated in WP in the comparisons WB5P vs. YB5P, WB7P vs. YB8P, WS1P vs. YS1P, and WS3P vs. YS3P, respectively (Fig. 3a; Supplementary Table S3). Venn diagram analysis revealed 1,209 DEGs that were common to all four comparison groups (Fig. 3b), indicating that these 1,209 genes were differentially expressed at all four stages between WP and ZS11 petals. GO and KEGG enrichment analysis (performed on the OmicShare online platform) classified the DEGs into 237 GO terms, with 725, 3,446, and 2,640 genes assigned to 16 terms in the cellular component category, 114 terms in the molecular function category, and 107 terms in the biological process category, respectively (Supplementary Table S4).

The most highly enriched terms in the cellular component category were membrane (GO:0016020) and photosystem (GO:0009521). The most highly enriched terms in the molecular function category were catalytic activity (GO:0,003,824), oxidoreductase activity (GO:0016491), and transferase activity (GO:0016740). The most highly enriched terms in the biological process category were metabolic process (GO:0008152), single-organism process (GO:0044699), single-organism metabolic process (GO:0044710), and oxidation–reduction process (GO:0055114). The top 50 most significantly enriched terms are shown in Supplementary Fig. S2. KEGG analysis revealed 46 enriched pathways. The most highly enriched pathways were metabolic pathways (ko01100), biosynthesis of secondary metabolites (ko01110), plant hormone signal transduction (ko04075), and carbon metabolism (ko01200). Most importantly, the flavonoid biosynthesis (ko00941), flavone and flavonol biosynthesis (ko00944), and carotenoid biosynthesis (ko00906) pathways were significantly enriched as well (Supplementary Fig. S3).

### Carotenoid metabolic pathway analysis

**Fig. 4:**
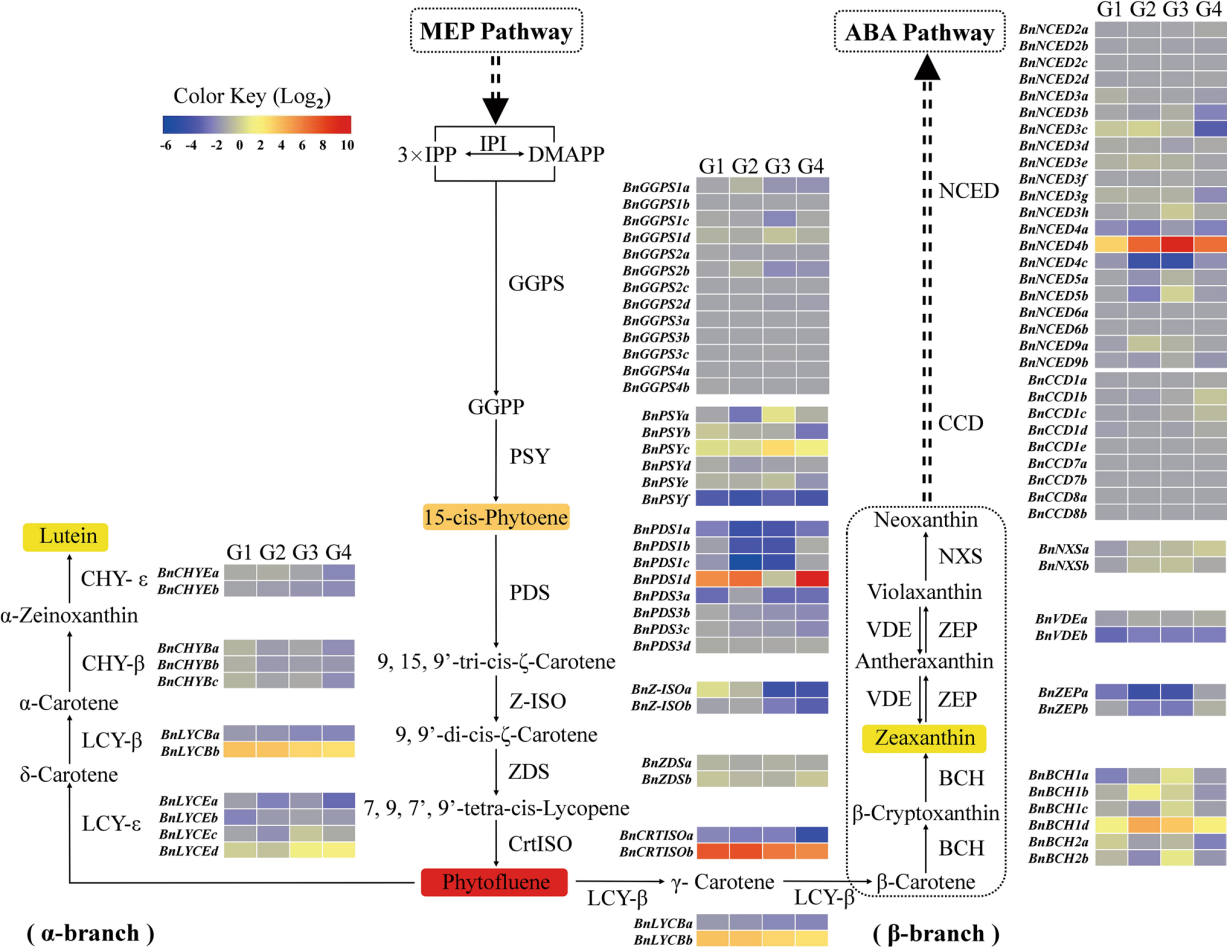
Diagram of the carotenoid metabolic pathway in WP and ZS11 petals. G1, G2, G3 and G4 indicate the four comparison groups: WB5P vs. YB5P, WB7P vs. YB8P, WS1P vs. YS1P and WS3P vs. YS3P, respectively. Fold changes (the mean of the three biological repeats for each sample) shown in color, yellow–red, gray and blue boxes indicate up-regulation, no obvious change and down-regulation of expression of the genes encoding these enzymes, respectively. Eighty-six carotenoid metabolism genes were identified in the *B*. *napus* genome, and the transcript levels are shown in Supplementary Fig. S4

The focus of this study was to investigate the differential accumulation of carotenoids in WP vs. ZS11 petals. Therefore, we examined the expression of 86 genes encoding proteins associated with carotenoid biosynthesis and degradation in the *B. napus* genome (Fig. 4; Supplementary Fig. S4). We found that 20 of these genes were significantly differentially expressed in WP vs. ZS11 petals during at least one stage of development (Supplementary Table S5; Supplementary Fig. S5).

Of the 86 genes in the carotenoid metabolic pathway, only 49 genes were obviously expressed during at least one stage in WP or ZS11 petals (Supplementary Fig. S4), with FPKM values > 5. Only two genes (*BnGGPS1a*, *BnGGPS1c*) in the carotenoid biosynthesis pathway were obviously expressed in WP and ZS11 petals, even though there are 13 homologous *GGPS* genes in the *B. napus* genome. However, five of six *PSY* genes (all except *BnPSYd*) were clearly expressed in both WP and ZS11 petals. One *PDS* gene (*BnPDS1d*), one *CrtISO* gene (*BnCrtISOb*), and one *LYCE* gene (*BnLYCEb*) were clearly expressed in WP petals but showed almost no expression in ZS11 petals. Importantly, the FPKM values of the two *VDE* genes (*BnVDEa* and *BnVDEb*) were < 3, indicating that these *VDE* genes, which participate in the xanthophyll cycle, were expressed at very low levels in both WP and ZS11 petals. These results indicate that little violaxanthin and antheraxanthin can be transformed into zeaxanthin (a reaction catalyzed by VDE) in these petals. Only nine of the 30 genes in the carotenoid degradation pathway were clearly expressed in WP and ZS11 petals during various stages of petal development, including 2 *CCD1* subfamily members (*BnCCD1b*, *BnCCD1c*), 2 *NCED3* subfamily members (*BnNCED3c*, *BnNCED3g*), 3 *NCED4* family members (*BnNCED4a*, *BnNCED4b*, *BnNCED4c*), and 2 *NCED5* subfamily members (*BnNCED5a*, *BnNCED5b*). However, members of the *BnCCD2*, *BnCCD7*, *BnCCD8*, *BnNCED6*, and *BnNCED9* subfamilies showed almost no expression in both WP and ZS11 petals, indicating that they did not function during petal development. These results provide an important reference for studying carotenoid metabolism in *B*. *napus* petals.

### qRT-PCR analysis of DEGs in the carotenoid metabolic pathway

**Fig. 5:**
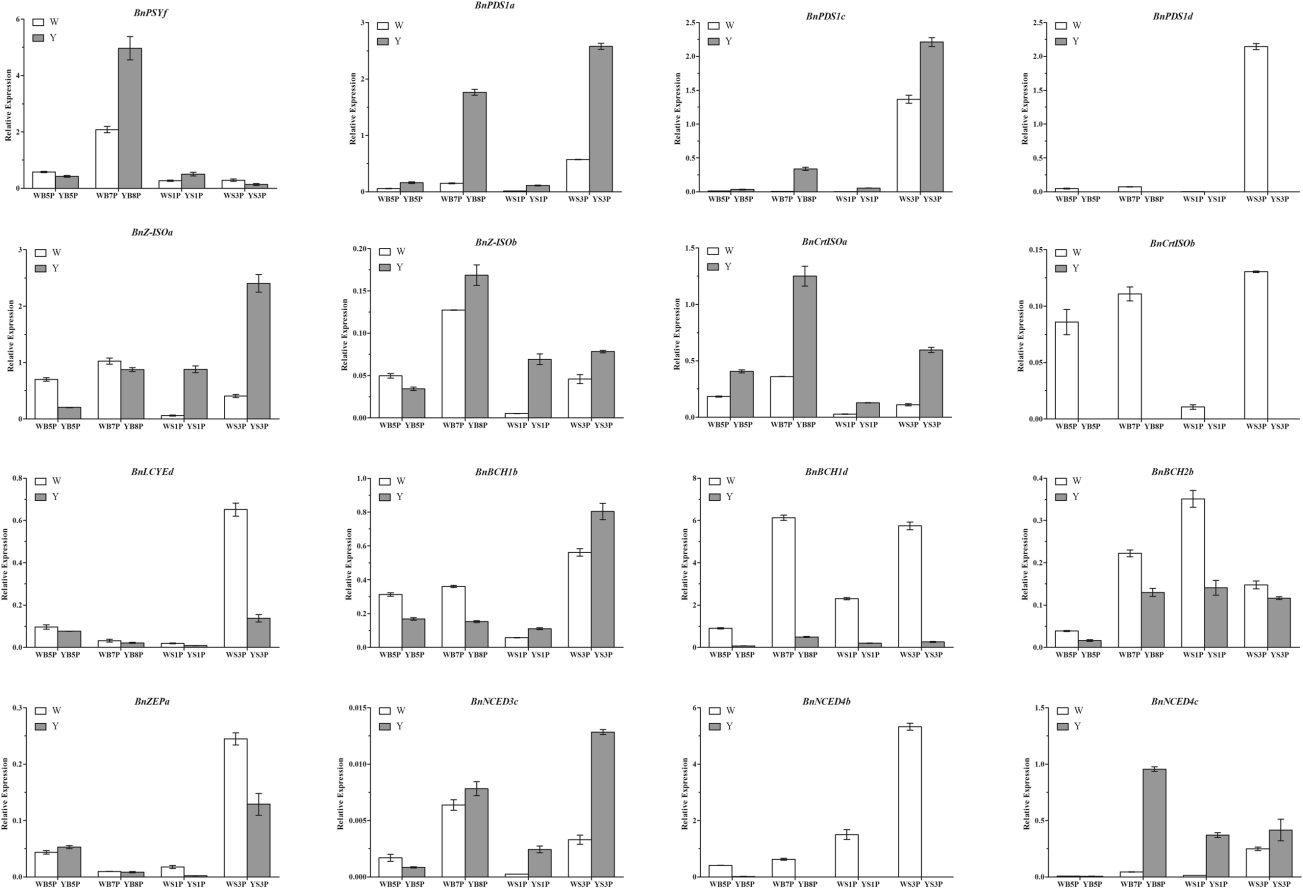
Relative expression of candidate DEGs in the carotenoid metabolic pathway determined by qRT-PCR. Twenty genes were selected to perform expression analysis by qRT-PCR in WP and ZS11 petals at the four stages. Sixteen genes were confirmed to have significantly differential expressions in WP and ZS11 petals at different stages. Values are means ± SD of three biological experiments. Error bars indicate SEs

To further confirm the results of RNA-seq, we measured the transcript levels of these DEGs in the carotenoid metabolic pathway in WP and ZS11 petals at four stages of development by qRT-PCR using the primers listed in Supplementary Table S6. The 13 DEGs involved in carotenoid biosynthesis and 3 DEGs involved in carotenoid degradation were differentially expressed in WP and ZS11 petals at different stages, including *BnPSYf*, *BnPDS1a*, *BnPDS1c*, *BnPDS1d*, *BnZ-ISOa*, *BnZ-ISOb*, *BnCrtISOa*, *BnCrtISOb*, *BnLCYEd*, *BnBCH1b*, *BnBCH1d*, *BnBCH2b*, *BnZEPa*, *BnNCED3c*, *BnNCED4b*, and *BnNCED4c* (Fig. 5).

Various genes in the carotenoid biosynthesis pathway were significantly downregulated in WP petals compared to ZS11, including the following: one *PSY* gene (*BnPSYf*) at stage B7, two *PDS* genes (*BnPDS1a* and *BnPDS1c*) at all four stages, two *Z-ISO* genes (*BnZ-ISOa* and *BnZ-ISOb*) at S1 and S3, one *CrtISO* gene (*BnCrtISOa*) at all four stages, and one *BCH* gene (*BnBCH1b*) at S1 and S3. Several of these genes were highly upregulated in WP vs. ZS11 petals, including one *PDS* gene (*BnPDS1d*) at S3, one *CrtISO* gene (*BnCrtISOb*) at all four stages, one *LCYE* gene (*BnLCYEd*) at S3, one *BCH* gene (*BnBCH1b*) at stages B5 and B7, two BCH genes (*BnBCH1d* and *BnBCH2b*) at all four stages, and one ZEP gene (BnZEPa) at S3. Among genes in the carotenoid degradation pathway, two *NCED* DEGs (*BnNCED3c* and *BnNCED4c*) were markedly downregulated in WP petals compared to ZS11, whereas only *BnNCED4b* was markedly upregulated in WP petals during all four stages of development.

### Transcription factors involved in regulating carotenoid metabolism

In addition to structural genes, transcription factors play vital roles in regulating the carotenoid metabolic pathway. Members of several transcription factor families, including the MYB, MADS-box, NAC, bHLH, SBP box, AP2/ERF, HD ZIP, NF-Y, and WRKY families, are the major regulators responsible for the transcriptional activation or repression of carotenoid metabolic genes (Stanley and Yuan 2019). The plant transcription factor database PlantTFDB is an excellent resource for functional and evolutionary analysis of plant transcription factors (Jin et al. 2016). Using PlantTFDB, we identified 489, 306, 411, 553, 63, 506, 178, 115, and 285 genes of the MYB, MADS-box, NAC, bHLH, SBP-box, AP2/ERF, HD-ZIP, NF-Y, and WRKY families, respectively, in the reference genome of *B. napus* cultivar ‘Darmor-*bzh*’. Among these, 52 BnMYB genes, 18 BnMADS genes, 55 BnNAC genes, 77 BnbHLH genes, 13 BnSBP genes, 88 BnAP2/ERF genes, 38 BnHD-ZIP genes, 14 BnNF-Y genes, and 27 BnWRKY genes were differentially expressed in WP vs. ZS11 petals at different stages of development (Supplementary Table S5).

**Fig. 6:**
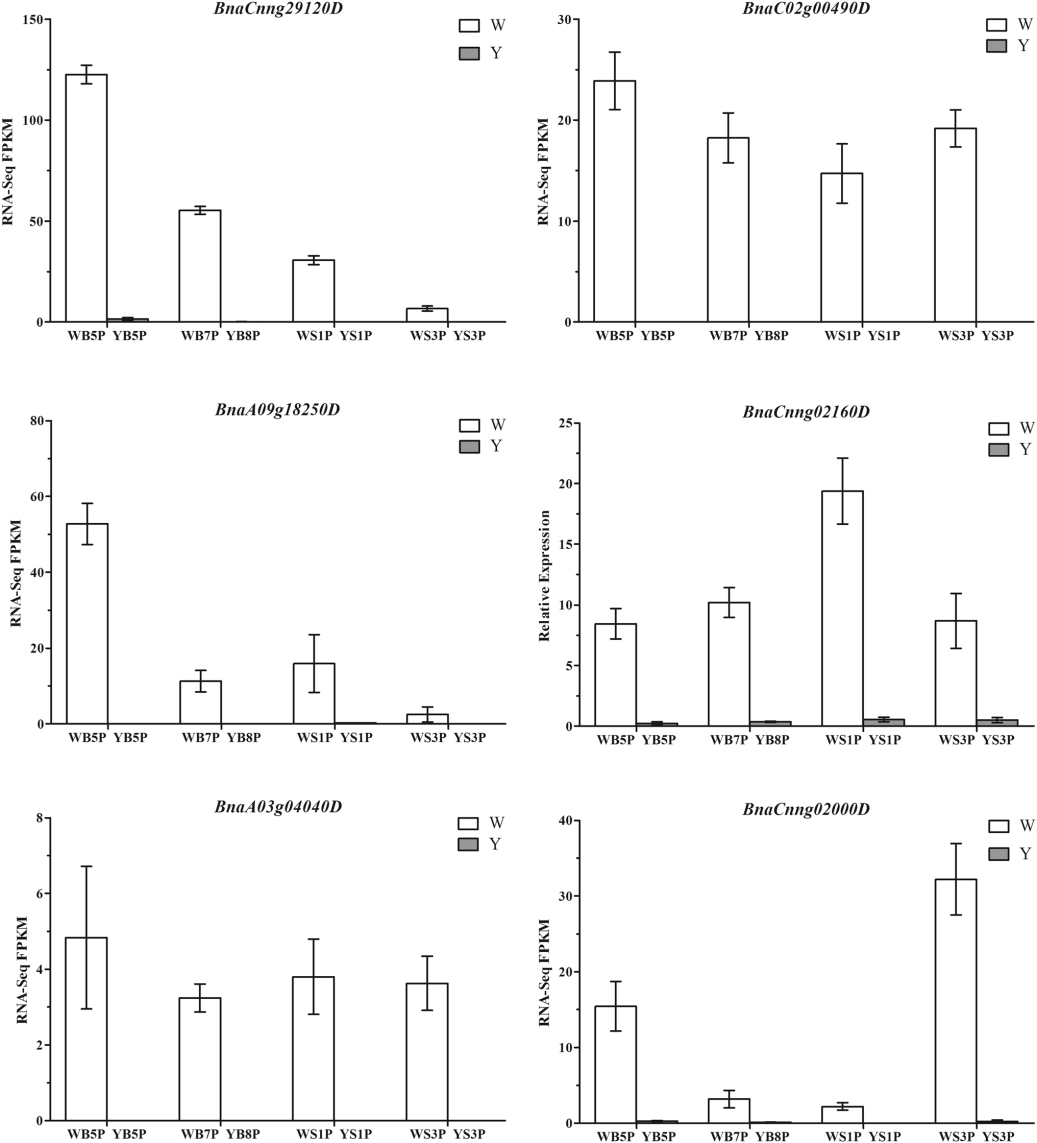
Transcript levels of candidate transcription factor genes for regulation of carotenoids. All six genes were up-regulated expressed significantly in WP petals at four stages compared with ZS11 petals. Values are means ± SD of three biological experiments. Error bars indicate SEs

Among the differentially expressed transcription factor genes, three BnMYB genes, two BnMADS-box genes, two BnNAC genes, four BnbHLH genes, one BnSBP-box gene, one BnAP2 gene, two BnHD-ZIP genes, three BnNF-Y genes, and one BnWRKY gene were dramatically differentially expressed in WP vs. ZS11 petals at all four stages of development (Supplementary Table S5). However, only one BnMYB106 gene (*BnaCnng29120D*), one BnMADS gene (*BnaC02g00490D*), two BnHD-ZIP genes (*BnaA09g18250D*, *BnaCnng02160D*), one BnNFYA1 gene (*BnaA03g04040D*), and one BnWRKY22 gene (*BnaCnng02000D*) were significantly upregulated in WP vs. ZS11 petals at all four stages of development (Fig. 6). These results are consistent with the expressive pattern of the candidate gene *BnNCED4b*, which was dramatically upregulated in WP petals and had almost no detectable expression in ZS11 petals.

## Discussion

Flavonoids and carotenoids are important yellow pigments in plants, but no significant difference in total flavonoid content was detected in white vs. yellow *B. napus* petals (Yin et al. 2019), indicating that different flavonoid contents are not the main factor causing petals to fade from yellow to white in line WP. In the current study, only ten of the 13 carotenoids examined were identified by metabolomic analysis in WP and ZS11 petals at S2 and S4, and no other products of intermediate metabolism were detected. Perhaps the unidentified carotenoids were present at extremely low levels below the detection limit. Most intermediate carotenoids contain multiple isomers, making it difficult to prepare or purchase standards, which hampers the detection and analysis of all intermediate metabolites in the carotenoid metabolic pathway.

We performed transcriptome analysis to investigate the molecular mechanism underlying petal color during petal development in *B. napus* lines WP and ZS11. We identified 1209 DEGs in four comparison groups at four stages of development. We focused on genes involved in carotenoid metabolism and identified 86 genes in the carotenoid metabolic pathway in the *B. napus* genome (Fig. 4; Supplementary Fig. S4), within the 49 genes obviously expressed during at least one stage in WP or ZS11 petals with FPKM values > 5, there were only 20 genes were differentially expressed in different comparison groups, which had not been reported previously. Moreover, we found three *ISO* genes (*BnZ-ISOa*, *BnZ-ISOa* and *BnCrtISOa*) were significantly downregulated in WP vs. ZS11 petals at S1, which may be related to the reduced total lutein and zeaxanthin contents in the white-flowered line. In the *crtiso* mutants of Chinese kale, the carotenoid isomerase gene (*BoaCRTISO*) was targeted and edited using the CRISPR/Cas9 system, the expression levels of *CRTISO* and most carotenoid and chlorophyll biosynthesis-related genes were notably lower than in the WT plants, in addition, both the total and individual carotenoid and chlorophyll concentrations were reduced, and the total levels declined by 11.89–36.33% (Sun et al. 2020). Most importantly, *BnNCED4b* was dramatically upregulated in WP petals and showed almost no expression in ZS11 petals, suggesting that this gene plays a key role in turning WP petals from pale yellow to white. This result is consistent with the previous finding that the carotenoid cleavage dioxygenase 4 gene *BnaC3.CCD4*, which is homologous to *BnNCED4b*, is responsible for the formation of flower color, with preferential expression in the petals of white-flowered *B*. *napus* lines (Zhang et al. 2015).

Based on the plant transcription factor database PlantTFDB (Jin et al. 2016), we identified 2906 transcription factor genes may be involved in the transcriptional activation or repression of carotenoid metabolism in *B. napus*. Of these, 382 genes were differentially expressed in different comparison groups, including 19 genes that were dramatically differentially expressed and six genes that were significantly upregulated in WP petals at all four stages of development compared to ZS11 petals. A comparison of these genes with the annotations of homologous genes in Arabidopsis indicated that *AtMYB106* (homologous to the BnMYB106 gene *BnaCnng29120D*) encodes a MIXTA-like transcription factor (NOECK) thought to negatively regulate trichome branching (Jakoby et al. 2008). The significantly upregulated BnMADS gene *BnaC02g00490D* is homologous to *AtFLC*, which encodes a key floral repressor in the vernalization pathways in Arabidopsis (Michaels and Amasino 1999). *AtHAT2*, which is homologous to the BnHD-ZIP gene *BnaA09g18250D*, plays opposite roles in regulating auxin-mediated morphogenesis in shoot and root tissues (Sawa et al. 2002). *AtANL2*, which is homologous to the BnHD-ZIP gene *BnaCnng02160D*, affects anthocyanin accumulation and root development (Kubo et al. 1999). The mutation of *AtNFYA1* (homologous to the BnNFYA1 gene *BnaA03g04040D*) led to defects in male gametogenesis and embryogenesis (Mu et al. 2013), and AtWRKY22 (homologous to the BnWRKY22 gene *BnaCnng02000D*) regulates the expression of senescence-associated genes indirectly or through other WRKY-mediated senescence signaling pathways (Zhou et al. 2011).

WRKY transcription factors recognize and bind to TTGAC(C/T) W-box *cis*-elements in the promoters of their target genes (Zhou et al. 2011). *OfWRKY3*, encoding a positive regulator of *OfCCD4*, is strongly expressed during the entire flowering season in the white-flowered *Osmanthus fragrans* variety ‘Zi Yingui’ and is barely expressed during stages S1-S4 in ‘Chenghong Dangui’, which has orange-red petals (Han et al. 2016). Therefore, BnWRKY22 may function as an upstream factor that regulates *BnNCED4b* expression to promote the degradation of carotenoids in petals, causing them to turn white. However, whether and how BnWRKY22 regulates petal color in *B. napus* via *BnNCED4b* is currently unknown. In summary, much work remains to be done to uncover the detailed molecular mechanisms by which *BnWRKY22* and *BnNCED4b* regulate color variation in petals by affecting the carotenoid metabolic pathway in *B*. *napus*.

## Conclusion

We combined metabolomic, transcriptomic, and qRT-PCR analyses to explore the mechanisms underlying the differential accumulation of carotenoids in WP and ZS11 petals. The decreased total lutein and zeaxanthin contents in WP petals cause them to fade from light yellow to white as the flowers mature. Transcriptome analysis revealed 20 DEGs in the carotenoid metabolism pathway. Of these, *BnNCED4b*, a gene involved in carotenoid degradation, represents a key candidate gene for the formation of white petals. The results of qRT-PCR analysis were consistent with the results of RNA-seq. Further analysis of transcription factor genes related to carotenoid accumulation suggested that BnWRKY22 might function upstream of *BnNCED4b* to promote the degradation of carotenoids in petals. These results provide important insights into the molecular mechanisms of the carotenoid metabolic pathway in *B*. *napus* petals. In addition, the candidate genes identified in this study provide a resource for the creation of new rapeseed materials with different flower colors.

### *Author contribution statement*

LDJ designed and performed all of the experiments and wrote the manuscript. XTZ, FYJ, and SSZ prepared the plant materials. MZD, CLQ, and XC performed RNA extraction. MCZ and GQM collected the phenotypic data in the field. JSW provided the WP material. CMQ and RW contributed to the experimental design. JSW and JNL approved of and modified the manuscript.

## Supplementary Information

Below is the link to the electronic supplementary material.

Supplementary file1 (DOCX 13 KB)

Supplementary file2 (DOCX 14 KB)

Supplementary file3 (DOCX 14 KB)

Supplementary file4 (DOCX 15 KB)

Supplementary file5 (DOCX 13 KB)

Supplementary file6 (TIF 31337 KB)

Supplementary file7 (TIF 100077 KB)

Supplementary file8 (TIF 106486 KB)

Supplementary file9 (TIF 119358 KB)

Supplementary file10 (TIF 118996 KB)

Supplementary file11 (XLSX 12 KB)

Supplementary file12 (XLSX 13 KB)

Supplementary file13 (XLSX 2026 KB)

Supplementary file14 (XLSX 87 KB)

Supplementary file15 (XLSX 16 KB)

Supplementary file16 (XLSX 11 KB)

Supplementary file17 (XLSX 127 KB)

## Abbreviations

CCD: Carotenoid cleavage dioxygenases
DEG: Diferentially expressed gene
GO: Gene ontology
KEGG: Kyoto Encyclopedia of Genes and Genomes
NCED: Nine-cis-epoxycarotenoid dioxygenases
