# Supplementary material for: Comparative transcriptomic and metabolomic analyses of carotenoid biosynthesis reveal the basis of white petal color in Brassica napus
Source: Planta. 2021 Jan 2;253(1):8. doi: 10.1007/s00425-020-03536-6 (PMC7778631; doi:10.1007/s00425-020-03536-6)
Supplement: Supplementary file 1 — Supplementary file1 (DOCX 13 KB) [file 425_2020_3536_MOESM1_ESM.docx]

**Supplementary Fig. S1** Major carotenoids in the petals of WP and ZS11 at S2 and S4 stages. The petal samples were collected from 30 randomly selected plants in the same block of WP and ZS11, and the metabolomic analysis of carotenoids was performed in a LC/MS/MS system. Among the 13 carotenoids examined, ten were identified in the samples
