# Supplementary material for: Comparative transcriptomic and metabolomic analyses of carotenoid biosynthesis reveal the basis of white petal color in Brassica napus
Source: Planta. 2021 Jan 2;253(1):8. doi: 10.1007/s00425-020-03536-6 (PMC7778631; doi:10.1007/s00425-020-03536-6)
Supplement: Supplementary file 2 — Supplementary file2 (DOCX 14 KB) [file 425_2020_3536_MOESM2_ESM.docx]

**Supplementary Fig. S2** GO enrichment analysis of the 10,116 DEGs in the four com parison groups in WP and ZS11 petals. GO terms with *P*-value < 0.05 were considered to be significantly enriched by the DEGs, and the enrichment results were plotted using online software available on OmicShare. The size of the dot represents the number of genes, and the color scale is based on *P* values of GO terms
