# Supplementary material for: Comparative transcriptomic and metabolomic analyses of carotenoid biosynthesis reveal the basis of white petal color in Brassica napus
Source: Planta. 2021 Jan 2;253(1):8. doi: 10.1007/s00425-020-03536-6 (PMC7778631; doi:10.1007/s00425-020-03536-6)
Supplement: Supplementary file 3 — Supplementary file3 (DOCX 14 KB) [file 425_2020_3536_MOESM3_ESM.docx]

**Supplementary Fig. S3** KEGG enrichment analysis of the DEGs in the four comparison groups in WP and ZS11 petals. KEGG pathways with *P*-value < 0.05 were considered to be significantly enriched by the DEGs, and the enrichment results were plotted using online software available on OmicShare. The size of the dot represents the number of genes, and the color scale is based on *P* values of KEGG pathways
