# Supplementary material for: Comparative transcriptomic and metabolomic analyses of carotenoid biosynthesis reveal the basis of white petal color in Brassica napus
Source: Planta. 2021 Jan 2;253(1):8. doi: 10.1007/s00425-020-03536-6 (PMC7778631; doi:10.1007/s00425-020-03536-6)
Supplement: Supplementary file 4 — Supplementary file4 (DOCX 15 KB) [file 425_2020_3536_MOESM4_ESM.docx]

**Supplementary Fig. S4** Transcript levels of genes in the carotenoid metabolic pathway in WP and ZS11 petals according to RNA-seq. 86 genes encoding proteins associated with carotenoid biosynthesis and degradation were identified in the *B. napus* genome, and the FPKM values of these genes are the means of three biological replications
