# Supplementary material for: Comparative transcriptomic and metabolomic analyses of carotenoid biosynthesis reveal the basis of white petal color in Brassica napus
Source: Planta. 2021 Jan 2;253(1):8. doi: 10.1007/s00425-020-03536-6 (PMC7778631; doi:10.1007/s00425-020-03536-6)
Supplement: Supplementary file 5 — Supplementary file5 (DOCX 13 KB) [file 425_2020_3536_MOESM5_ESM.docx]

**Supplementary Fig. S5** Transcript levels of the twenty DEGs in carotenoid metabolic pathway according to RNA-seq. All twenty genes were significantly differentially expressed in WP vs. ZS11 petals during at least one stage of development. Values are means ± SD of three biological experiments. Error bars indicate SEs
