# Supplementary figures and images for: Comparative transcriptomic and metabolomic analyses of carotenoid biosynthesis reveal the basis of white petal color in Brassica napus
Source: Planta. 2021 Jan 2;253(1):8. doi: 10.1007/s00425-020-03536-6 (PMC7778631; doi:10.1007/s00425-020-03536-6)

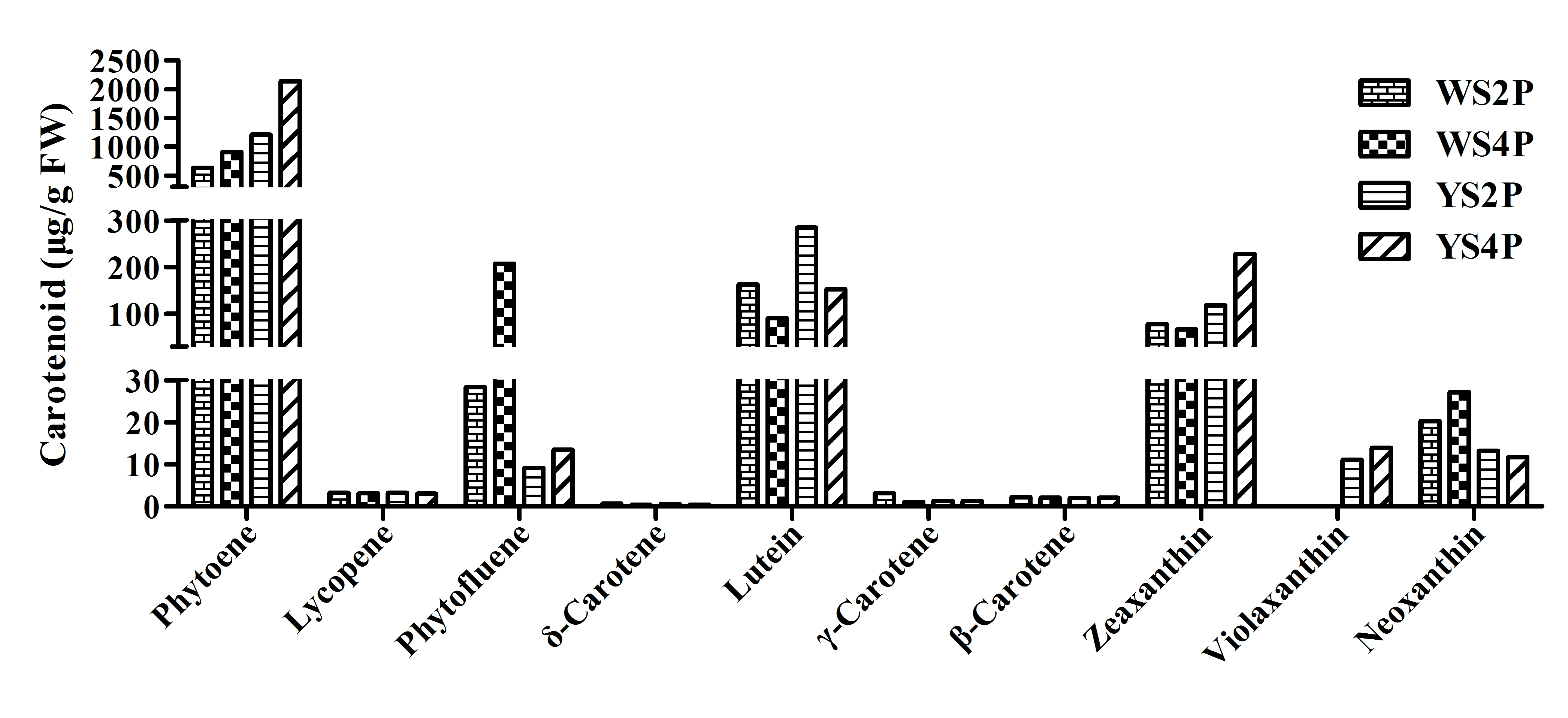

Supplement: Supplementary file 6 — Supplementary file6 (TIF 31337 KB) [file 425_2020_3536_MOESM6_ESM.tif]
